# Mechanistic Studies of Homo- and Heterodinuclear Zinc Phosphoesterase Mimics: What Has Been Learned?

**DOI:** 10.3389/fchem.2019.00082

**Published:** 2019-02-21

**Authors:** Andrea Erxleben

**Affiliations:** School of Chemistry, National University of Ireland Galway, Galway, Ireland

**Keywords:** zinc, hydrolysis (esters), phosphatase, biomimicry, catalysis

## Abstract

Phosphoesterases hydrolyze the phosphorus oxygen bond of phosphomono-, di- or triesters and are involved in various important biological processes. Carboxylate and/or hydroxido-bridged dizinc(II) sites are a widespread structural motif in this enzyme class. Much effort has been invested to unravel the mechanistic features that provide the enormous rate accelerations observed for enzymatic phosphate ester hydrolysis and much has been learned by using simple low-molecular-weight model systems for the biological dizinc(II) sites. This review summarizes the knowledge and mechanistic understanding of phosphoesterases that has been gained from biomimetic dizinc(II) complexes, showing the power as well as the limitations of model studies.

## Introduction

The hydrolytic cleavage of phosphate esters is an important biochemical reaction in living systems, playing a fundamental role in energy metabolism (Berg et al., 2010), DNA repair (Cowan, 1998), RNA splicing (Kuimelis and McLaughlin, 1998), and signaling (Berg et al., 2010). It is relevant to the breaking down of bone material by osteoclasts (bone resorbing cells) in mammals and to the absorption and mobilization of phosphorus in plants (Cashikar et al., 1997; Oddie et al., 2000; Cleland and Hengge, 2006; Mitić et al., 2006; Schenk et al., 2013; Daumann et al., 2014). In certain bacteria phosphotriesterases have evolved that can hydrolyze organophosphates including insecticides and chemical warfare agents (Donarski et al., 1989; Dumas et al., 1990).

**Figure 1:**
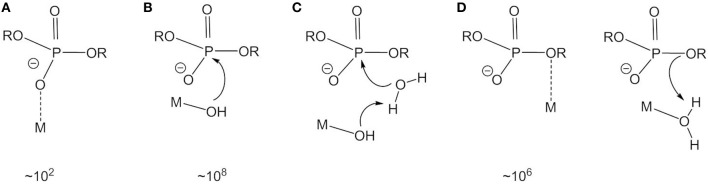
Activation modes and rate accelerations for metal-catalyzed phosphate ester hydrolysis. **(A)** Lewis acid activation. **(B)** Nucleophile activation. **(C)** Base catalysis. **(D)** Leaving group stabilization.

Under physiological conditions, phosphate esters are highly resistant toward hydrolysis (Cleland and Hengge, 2006). The half-life of a phosphodiester bond in the backbone of DNA has been estimated to be on the order of hundreds to thousands of millions of years (Williams et al., 1999; Schroeder et al., 2006). Yet DNAses can cleave DNA within seconds to minutes (Cowan, 1998). The majority of enzymes that catalyze phosphate ester hydrolysis contain two or more metal ions in their active site. Zn^2+^, which is a strong Lewis acid, labile and not redox active, is ideally suited for biological hydrolysis reactions. The use of metal complexes that mimic the structure and function of a metalloenzyme is a well-established approach in bioinorganic chemistry to develop highly effective catalysts modeled after nature and to gain a molecular level understanding of the enzymatic mechanism. In the late 1970s and 1980s pioneering work by the groups of Sargeson (Anderson et al., 1977; Jones et al., 1983; Hendry and Sargeson, 1989), Breslow (Gellman et al., 1986; Breslow et al., 1989), and Chin (Chin, 1991) among others gave the first insight into the role of the metal ion(s) in the mechanisms of phosphoester hydrolysis by metallohydrolases. Using phosphate esters with good leaving groups and kinetically inert mononuclear Co(III) complexes, metal-catalyzed hydrolysis reactions were shown to proceed through the following mechanisms: (i) Lewis acid activation, in which the metal polarizes the P-O bond and activates the phosphorus for nucleophilic attack (Figure 1A); (ii) metal hydroxide activation, in which the metal generates (metal-bound) hydroxide to act as an efficient nucleophile at pH 7 or as a general base (Figures 1B,C); (iii) stabilization of the leaving group (Figure 1D); and (iv) combinations of (i), (ii), and (iii). Mechanistic information was obtained through detailed kinetic studies including the measurement of rate-pH profiles and kinetic isotope effects. The rate accelerations achieved by the different activation modes could be quantified (Williams et al., 1999). Kimura and coworkers used macrocyclic dinuclear Zn(II) complexes to study the relationship between the number and type of donor atoms and the catalytic efficiency (Koike and Kimura, 1991). Later, the work was extended to dinuclear complexes that model the cooperativity of the metal ions in bimetallic hydrolases and to metal complexes, with pendant functional groups to mimic secondary interactions between the substrate and amino acid side chains in the active site of metalloenzymes (Young and Chin, 1995; Kimura, 2000; Daumann et al., 2014). A lot of what has been learned through the early studies has informed the rational design of highly efficient catalysts, often with non-biological metals such as lanthanides (Franklin, 2001; Liu and Wang, 2009). Metal complex-based hydrolysis catalysts have been discussed in several excellent review articles (Franklin, 2001; Mancin and Tecilla, 2007; Liu and Wang, 2009; Desbouis et al., 2012; Yu and Cowan, 2018).

The increasing role of computational chemistry has led to a renewed interest in mechanistic questions and a significant number of theoretical and combined theoretical and experimental studies have been published that investigated the mechanistic pathways in detail. This review intends to give a concise account of the contribution of experimental and computational studies of dinuclear biomimetic zinc(II) complexes to our current understanding of the mechanistic details of enzymatic phosphate ester hydrolysis with a focus on the recent literature.

## Phosphomonoester Hydrolysis

The half-life for the spontaneous hydrolysis of dianionic phosphomonoesters, ROP(O)${}_{3}^{2-}$,

$$\begin{array}{l} ROP{\left(O\right)}_{3}^{2-}+H_{2}O\to HPO_{4}^{2-}+ROH \end{array}$$

is on the order of 10^12^ years at ambient temperature (Lad et al., 2002). In principle the reaction can proceed through different mechanisms; a dissociative mechanism involving a ${\text{PO}}_{3}^{-}$ intermediate (D_N_ + A_N_), an associative mechanism with a five-coordinate phosphorane intermediate (A_N_ + D_N_) or a concerted mechanism (A_N_D_N_) with an associative or dissociative transition state depending on the synchronicity of bond formation and departure of the leaving group.

**Figure 2:**
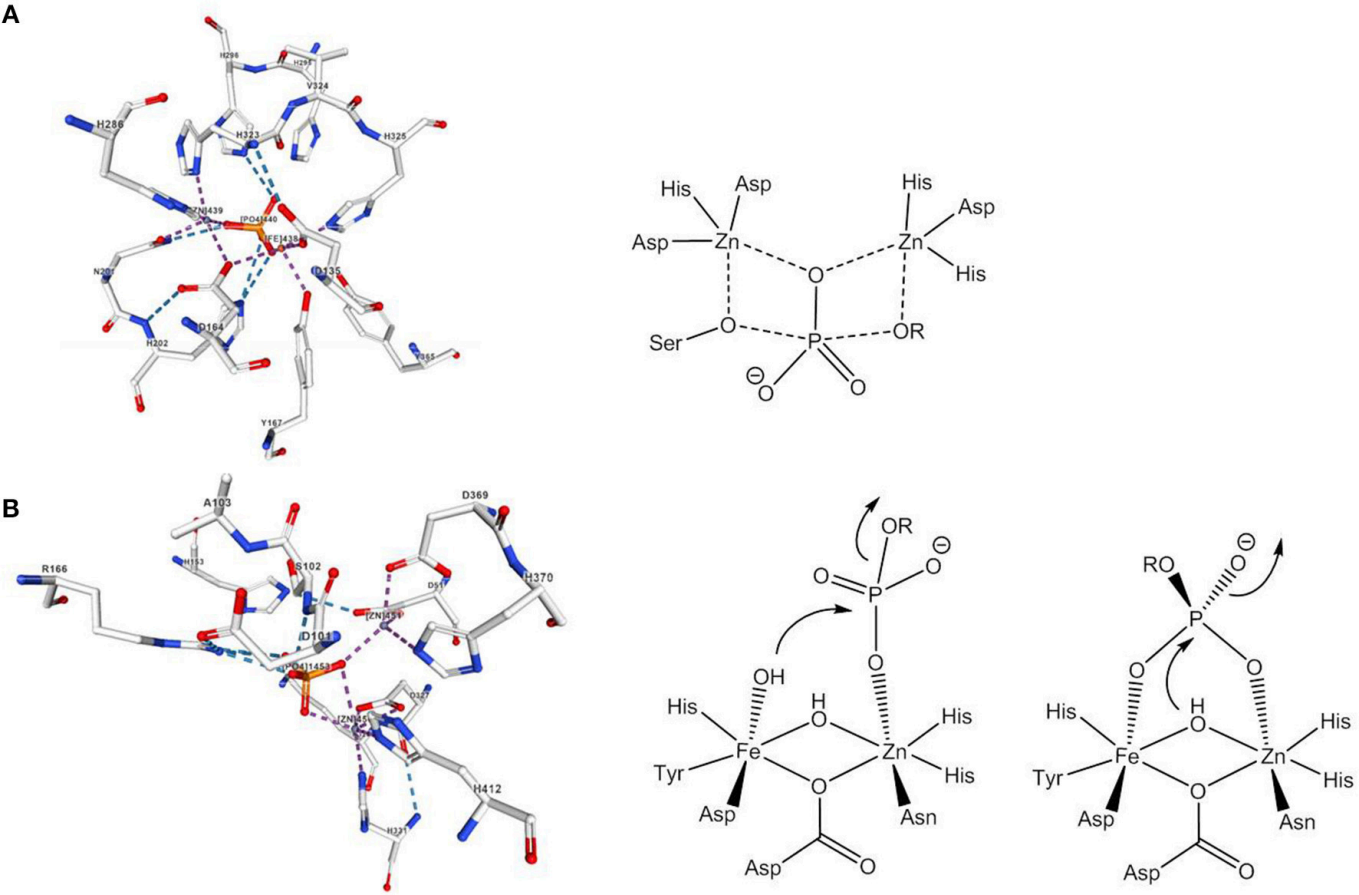
Active site and mechanism(s) of **(A)** *E. coli* alkaline phosphatase and **(B)** red kidney bean PAP. The image of the dizinc(II) site complex with phosphate in **(A)** was created with the PDB 1KHL and associated publication (Le Du et al., 2002). The image of the active site complex with phosphate in **(B)** was created with the PDB 4KBP and associated publication (Klabunde et al., 1996). NGL Viewer (Rose et al., 2018) and RCSB PDB.

In nature, the hydrolysis of phosphomonoesters is catalyzed by phosphomonoesterases such as alkaline phosphatase, purple acid phosphatase or inositol monophosphatase. The active site of alkaline phosphatase from *E. coli* contains two Zn^2+^ ions and a Mg^2+^ ion (Le Du et al., 2002). One of the phosphoryl oxygens is coordinated by the two Zn^2+^ ions, which also bind the nucleophile, a deprotonated serine, and the leaving group, respectively (Figure 2A). Experimental and theoretical data agree with a dissociative mechanism (Zalatan et al., 2007; López-Canut et al., 2009). Probably the best studied phosphomonoesterases are purple acid phosphatases (PAPs). PAPs are non-specific hydrolases that cleave a variety of phosphate esters and anhydrides at acidic pH. They contain a heterodinuclear Fe(III)-M(II) site and their characteristic purple color is due to a tyrosinate-to-Fe(III) ligand-to-metal charge transfer at about 560 nm (Mitić et al., 2006). The active site of red kidney bean PAP in which the divalent metal ion is Zn(II) (Sträter et al., 1995) is shown in Figure 2B. Although the sequence homology between PAPs from different sources is low, the seven amino acids that constitute the primary coordination sphere of the Fe(III)-M(II) core are conserved in all PAPs. The mechanism proposed by Klabunde et al. involves the monodentate coordination of the phosphate ester to the divalent metal ion followed by nucleophilic attack by Fe(III)-bound hydroxide (Klabunde et al., 1996). The strong Lewis acidity of Fe(III) allows the formation of Fe(III)-OH at acidic pH. For sweet potato PAP an alternative mechanism with bridging phosphate ester coordination and nucleophilic attack by a μ-(hydr)oxide was suggested (Schenk et al., 2005). Ga(III) can replace Fe(III) in the active site and studies indicated that PAPs can switch between the two mechanisms depending on the metal ion composition/availability/solubility, the second coordination sphere, the actual substrate, and the pH value (“one enzyme–two mechanisms” hypothesis; Mitić et al., 2006; Smith et al., 2007). While a bridging oxide would be an efficient nucleophile, the nucleophilicity of a hydroxide that is tightly bound to two metals should be rather low. It was therefore suggested that the bridging hydroxide in hydrolytic enzymes shifts to a (*pseudo*-)terminal position on binding of the substrate (Bennett and Holz, 1997; Wang et al., 1999). Computational evidence for such a μ-OH shift was seen in model systems for phosphodiesterases and will be discussed in the next section.

In contrast to the large number of studies on the catalysis of phosphodiester hydrolysis, the cleavage mechanism of phosphomonoesters by biomimetic zinc(II) complexes is little investigated (Anbu et al., 2012; Zhang et al., 2014a,c; Sanyal et al., 2015). In recent years, various studies have been aimed at elucidating the role of the heterodimetallic Fe(III)-Zn(II) site in PAP. However, the substrate employed is generally the phosphodiester bis (2,4-dinitrophenyl) phosphate (BDNPP), a widely used model for the phosphodiester linkages in DNA. Heterodinuclear Fe(III)-Zn(II) biomimetics that mostly do not show monophosphatase activity will therefore be discussed in the section on phosphodiester hydrolysis.

**Figure 3:**
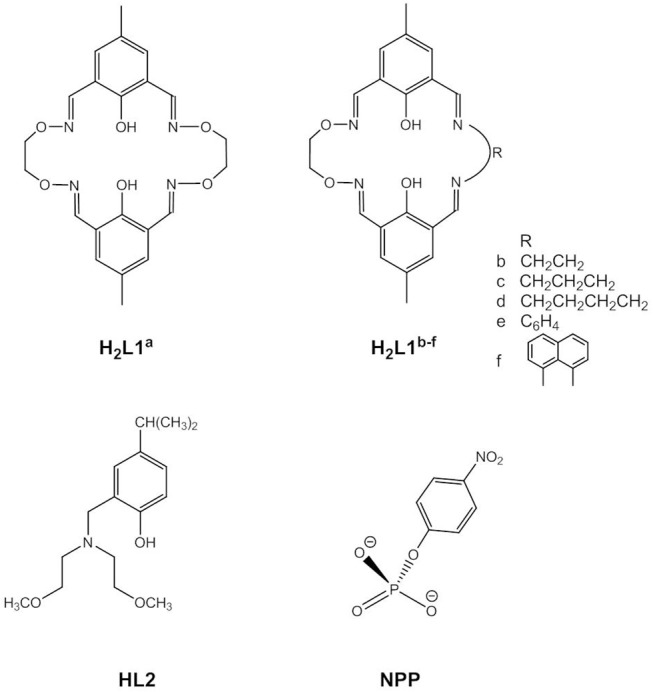
Substrate (NPP) and ligands in dinuclear Zn(II) complexes used to study phosphomonoester hydrolysis.

Phosphomonoester hydrolysis by dizinc(II) complexes is usually studied using 4-nitrophenyl phosphate as an ester with a good leaving group (NPP, Figure 3). The mechanism of the hydrolysis of the NPP^2−^ dianion is generally believed to be concerted with a loose transition state, while for phosphomonoester monoanions a dissociative mechanism involving metaphosphate as the intermediate has not been ruled out (Cleland and Hengge, 2006; Klähn et al., 2006; Kamerlin and Wilkie, 2007; Zhang et al., 2014a,c; Sanyal et al., 2015). The dianion is less reactive than the monoanion due to the higher negative charge of the transition state.

**Figure 4:**
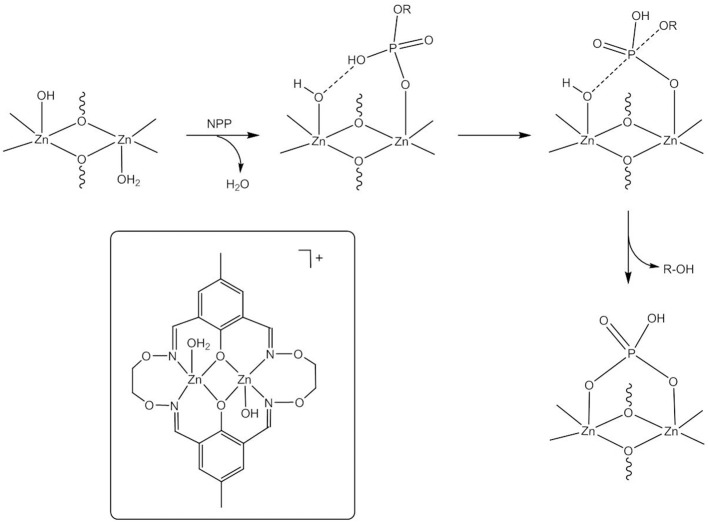
Catalytic species and proposed mechanism for NPP^−^ hydrolysis by the dizinc(II) complex of **H**_**2**_**L1**^**a**^.

Kandaswamy and coworkers synthesized dizinc(II) complexes with a series of dinucleating, oxyimine-based macrocyclic ligands (Anbu et al., 2012, Figure 3). The dizinc complex of the symmetric ligand **H**_**2**_**L1**^**a**^ that had the shortest Zn(II)^…^Zn(II) distance and the least distorted geometry hydrolyzed monoanionic NPP^−^ with a higher k_cat_ value than did the analogous complexes of unsymmetric **H**_**2**_**L1**^**b−f**^. The reaction kinetics showed a change in the reaction order at higher complex concentrations. Zhao and coworkers carried out DFT calculations to investigate the reaction mechanism (Zhang et al., 2014a,c). Different competitive catalytic mechanisms were found, depending on the concentration of the complex. At high concentrations two dinuclear entities form a hydroxido-bridged dimer that binds NPP^−^ to give the catalyst-substrate complex. Substrate coordination to two dizinc(II) entities is also favored in the medium concentration range. More relevant to the enzymatic reaction, at low concentrations NPP^−^ binds in a monodentate fashion to the catalytic species *trans*-[Zn_2_**L1**^**a**^(H_2_O)(OH)]^+^ followed by nucleophilic attack by Zn-OH to give a distorted trigonal bipyramidal phosphorane transition state (Figure 4). Substrate binding is stabilized by hydrogen bonding between the P-OH proton and Zn-OH. Cleavage of the P-O bond to the leaving group and protonation of the leaving group oxygen occur concurrently. In the case of unsymmetrical *trans*-[Zn_2_**L1**^**f**^(H_2_O)(OH)]^+^ hydrogen bonding between Zn-OH and P = O and between P-OH and Zn-OH exists in the catalyst-substrate complex (Zhang et al., 2014c). Again, the theoretical calculations indicated a concerted mechanism involving simultaneous bond formation to the nucleophile and breaking of the bond to the leaving group in the transition state. The P-OH proton forms a H-bond with the leaving group oxygen in the transition state and proton transfer and P-O bond cleavage are simultaneous. Modeling of a transition state with one phosphoryl atom coordinated to both Zn(II) centers of Zn**L1**^**f**^ and one Zn(II) additionally binding to the leaving group oxygen demonstrated that metal-induced leaving group activation is less favorable than proton transfer-assisted leaving group departure. In the calculated mechanisms NPP^−^ binds to the Zn(II) in the imine site, while the Zn(II) in the oxyimine site provides the nucleophile. The authors argued that the more electronegative oxygen atoms next to the imine nitrogens strengthen the Zn-N bonds and weaken the bond to the nucleophile. However, the X-ray structure of [Zn_2_**L1**^**c**^(H_2_O)_2_](ClO_4_)${}_{2}^{.}$3H_2_O revealed no significant differences in the Zn-N and Zn-OH_2_ bond lengths between both binding sites. Furthermore, other studies led to the opposite conclusion that electron-withdrawing substituents result in a stronger M-OH bond and thus decrease the nucleophilicity of metal-bound hydroxide by increasing the Lewis acidity of the metal ion (Coleman et al., 2010).

In the 2:2 complexes [Zn_2_**L2**_2_X_2_] (X = Cl, Br, I) two Zn(II)**L2** entities are linked through two phenoxide bridges. X-ray analysis revealed the expected *trans* orientation of the two halides and Zn(II)^…^Zn(II) distances around 3.1 Å, i.e., close to the metal-metal distance in zinc hydrolases (Sanyal et al., 2015). The phosphatase activity toward NPP^2−^ was studied in aqueous DMF, although it is not clear if the dinuclear structure is retained in solution. Theoretical calculations were described in the same paper and suggested that the D-*cis* form of the dinuclear complex is slightly more catalytically favorable than the D-*trans* form. In contrast to the macrocyclic complexes, a concerted reaction mechanism involving bidentate coordination of the phosphomonoester to both Zn(II) was found to be most favorable. One of the phenoxide bridges is replaced with a hydroxide so that Zn retains the more stable five-coordinate geometry. This bridging hydroxide serves as the nucleophile as proposed for sweet potato PAP (Figure 2B).

## Phosphodiester Hydrolysis

### Hydrolysis of DNA Model Substrates

**Figure 5:**
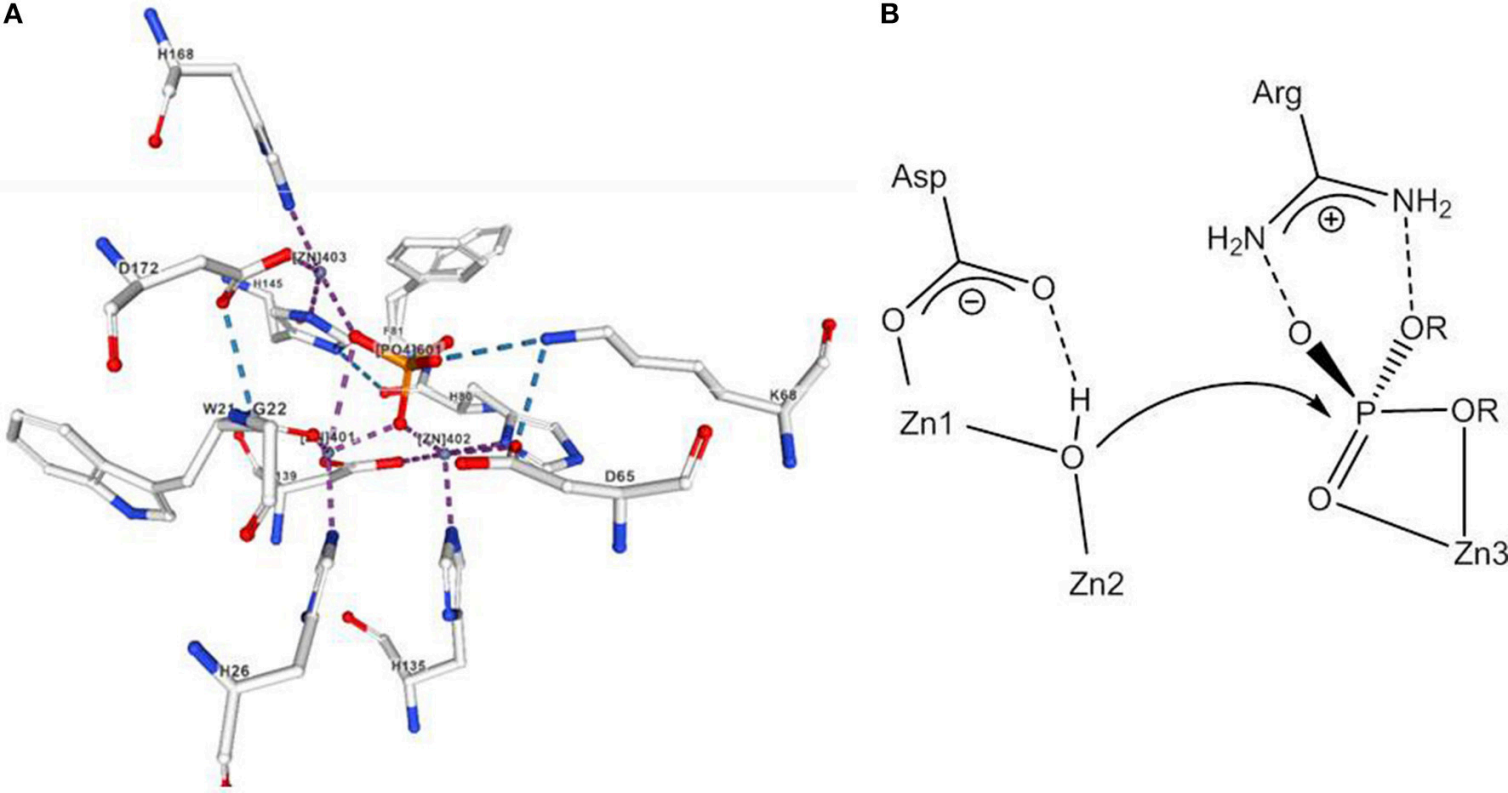
**(A)** Active site of nuclease from Aspergillus oryzae with a coordinated phosphate. **(B)** Mechanism of phosphodiester hydrolysis catalyzed by P1 nuclease. The image in **(A)** was created with the PDB 5FBA and associated publication (Koval et al., 2016). NGL Viewer (Rose et al., 2018) and RCSB PDB.

It is assumed that the uncatalyzed hydrolysis of phosphodiesters proceeds via a concerted mechanism with a loose transition state (Hengge, 2002). An example for a Zn(II) containing phosphodiesterase is P1 nuclease that cleaves single-stranded RNA and DNA into mononucleotides. P1 nuclease has a trimetallic active site; Zn3 binds to the phosphodiester group, while a hydroxide that bridges Zn1 and Zn2 at a distance of 3.2 Å is believed to act as the nucleophile (Volbeda et al., 1991; Figure 5).

**Figure 6:**
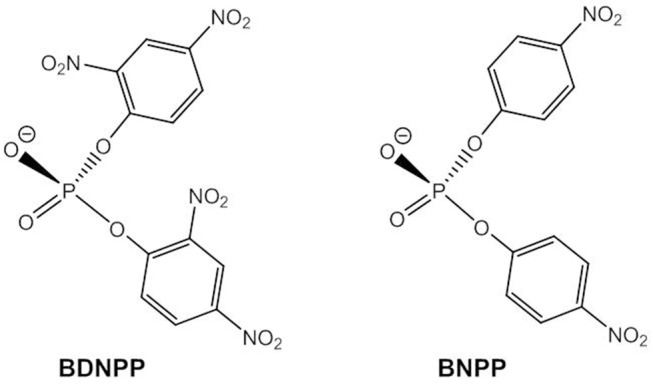
Chemical structures of the DNA models BDNPP and BNPP.

Many phosphodiesterase mimics have been designed with bridging acetate ligands and it is generally assumed that these are substituted by terminal and/or bridging hydroxide ligands in aqueous solution. It has also been shown that phosphodiesters can readily replace carboxylate ligands in dizinc(II) complexes (Daumann et al., 2013). The most popular models for the phosphodiester linkages in DNA are bis(2,4-dinitrophenyl) phosphate, BDNPP, and bis(4-nitrophenyl) phosphate, BNPP, (Figure 6) that are usually converted to 2,4-dinitrophenyl phosphate and 4-nitrophenyl phosphate without further hydrolysis of the respective monoester taking place.

**Figure 7:**
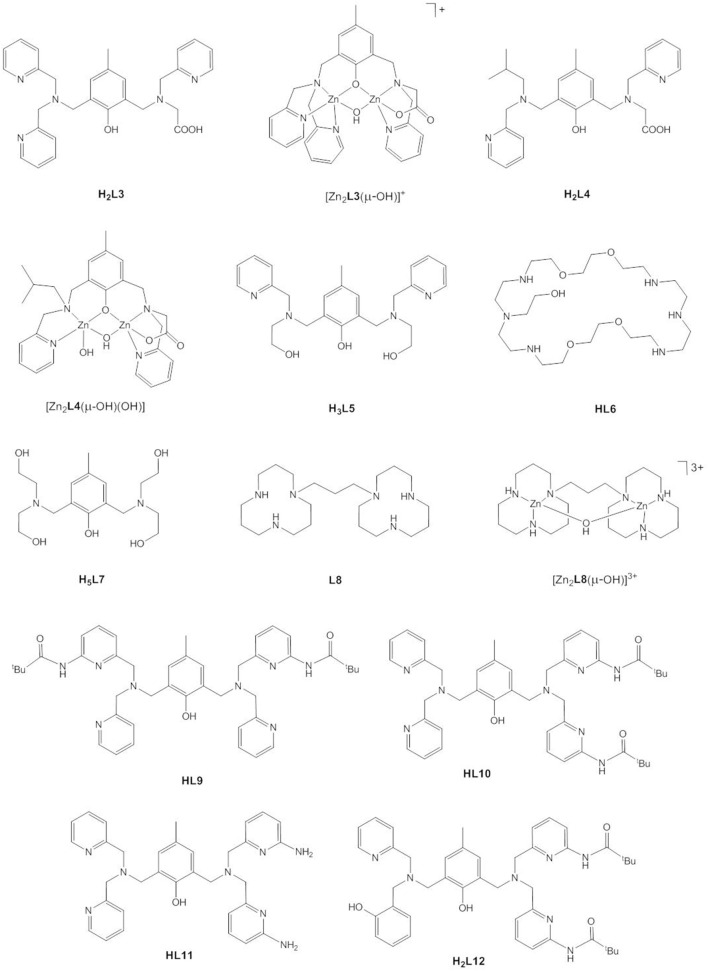
Chemical structures of ligands **H**_**2**_**L3** – **H**_**2**_**L12** and the dinuclear Zn(II) complexes [Zn_2_**L3**(μ-OH)]^+^, [Zn_2_**L4**(μ-OH)(OH)], and [Zn_2_**L8**(μ-OH)]^3+^.

Based on kinetic data, X-ray analysis of the complex cocrystallized with a phosphodiester and binding studies, the following mechanisms have been assigned to dinuclear zinc(II) catalysts; (i) monodentate coordination of the phosphodiester to one Zn(II) and nucleophilic attack by OH bound to the other Zn(II) (Bazzicalupi et al., 2004; Jarenmark et al., 2010; Pathak et al., 2018) or to the same Zn(II) (Massoud et al., 2016); (ii) nucleophilic attack by Zn-OH on the bridging substrate (Bazzicalupi et al., 1997, 2004; Daumann et al., 2012, 2013; Brown et al., 2016) and (iii) nucleophilic attack of the bridging substrate by a bridging hydroxide (Das et al., 2014, 2018; Montagner et al., 2014; Daver et al., 2016). As discussed above, a shift of μ-OH to a terminal position in mechanism (iii) would render the attacking hydroxide a better nucleophile. Das et al. carried out DFT calculations on the hydrolysis of BDNPP by the unsymmetric dinuclear Zn(II) complex [Zn_2_**L3**(μ-OH)]^+^ (Figure 7) which indicated that in the first step the phosphodiester binds to Zn1 in the *N*_3_*O*_2_ site followed by a concerted step with a transition state in which μ-OH is shifted toward Zn1 and the substrate adopts a bridging coordination mode (Das et al., 2014). DFT studies on the dizinc(II) complex of an analogous *N*_5_*O*_2_ ligand containing two 1-methylimidazole moieties (Das et al., 2018) and on the related unsymmetric dizinc(II) complex [Zn_2_**L4**(μ-OH)(OH)] found the same mechanism (Daver et al., 2016). By contrast, DFT calculations of the hydrolysis of BNPP by *trans*-[Zn_2_(**L1**^**a**^)(H_2_O)(OH)]^+^ suggested a stepwise mechanism involving nucleophilic attack by a terminally Zn-bound hydroxide and formation of the phosphorane intermediate as the rate-determining step (Zhang et al., 2014b). In the calculated mechanism bridging substrate binding also takes place in a stepwise manner with the phosphodiester binding initially via one phosphoryl oxygen to one Zn(II), followed by the formation of a second coordination bond between the nucleophile-binding Zn(II) and the other phosphoryl oxygen. This pathway appears to be favored over a concerted mechanism and over bridging OH acting as the nucleophile. It was noted that the macrocyclic ligand provides a rigid coordination sphere for the dizinc(II) site and imposes a relatively fixed Zn(II)^…^Zn(II) distance of 3.047 Å, close to the distance between the two phosphoryl oxygens in a phosphodiester (ca. 2.7 Å), which of course should affect the preferred mechanistic pathway.

**Figure 8:**
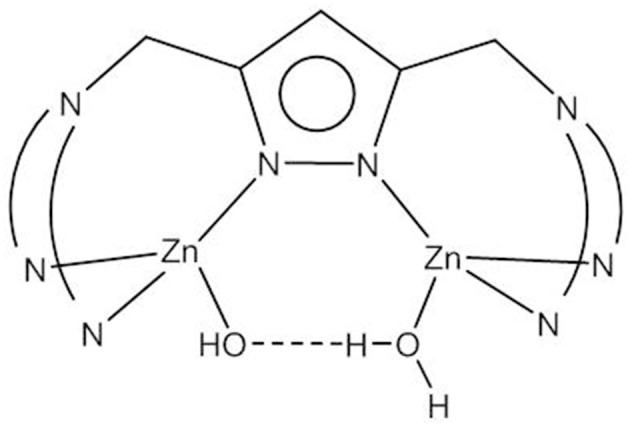
Pyrazolate-bridged dizinc(II) complexes with the Zn-O_2_H_3_-Zn motif.

The ability to provide a (metal-bound) hydroxide at physiological pH value is obviously a key feature of metallophosphatases—or in fact of any hydrolytic metalloenzyme. Binding to two Zn^2+^ ions in dinuclear model systems can decrease the pK_a_ of the Zn-bound water to below 8; however, as discussed above, a bridging coordination mode of the hydroxide is detrimental to its nucleophilicity. Meyer and coworkers developed a class of highly preorganized pyrazolate-based dizinc(II) complexes that allowed the systematic variation of the Zn(II)^…^Zn(II) distance (Bauer-Siebenlist et al., 2005; Meyer, 2006). By choosing the appropriate side arms, a large Zn(II)^…^Zn(II) separation could be enforced that accommodated a Zn-(H)O^…^HO(H)-Zn motif in which a Zn-bound hydroxide is held by strong hydrogen bonding in an intramolecular O_2_H_3_ bridge (Figure 8). It was shown that the formation of the Zn-(H)O^…^HO(H)-Zn unit brings about a similar decrease in the pK_a_ of Zn-OH_2_ to around the physiological pH as does the formation of the tightly bridged Zn-(μ-OH_2_)-Zn motif.

**Figure 9:**
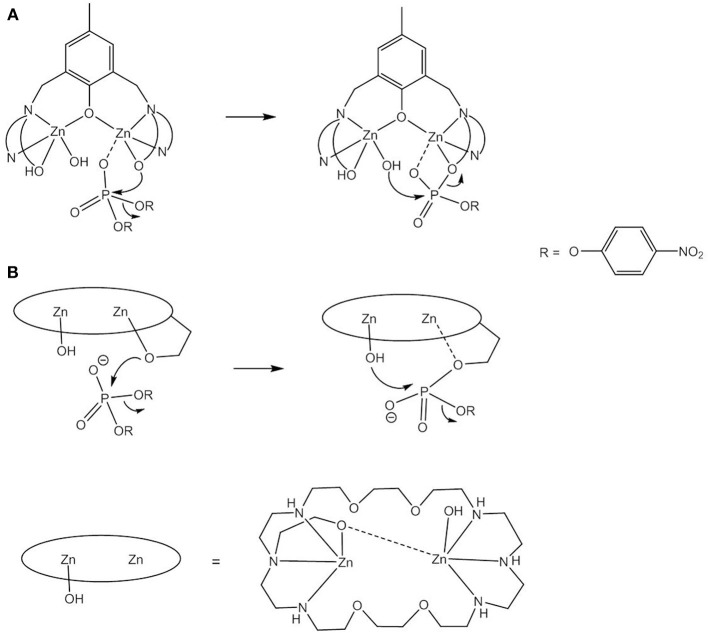
Sequential involvement of a Zn-OR and Zn-OH nucleophile in the cleavage of BNPP by [Zn_2_**HL5**]^2+^**(A)** and [Zn_2_**L6**(OH)]^2+^ **(B)**.

Another question addressed in model studies concerns the role of Zn-alkoxide. In some metallohydrolases, alcohol moieties are involved in the enzymatic mechanism (Weston, 2005). An example is alkaline phosphatase, whose two Zn^2+^ ions bind a phosphate ester in a bridging mode which is then nucleophilically attacked by a serine alcoholate. In the next step the P-O bond of the phosphorylated serine intermediate is cleaved following nucleophilic attack by a Zn-bound hydroxide. Alkaline phosphatase catalyzes the hydrolysis of phosphomonoesters under basic conditions. However, model studies were carried out with BDNPP and are therefore discussed in this section. For mononuclear Zn(II) complexes it has been shown that a coordinated alcohol is a better nucleophile than a coordinated water (Koike et al., 1995; Xia et al., 2003; Livieri et al., 2004). On this basis, Chen et al. proposed a mechanism involving nucleophilic attack by a Zn-bound alcoholate for the reaction of [Zn_2_**HL5**]^2+^, with BNPP giving a “transition complex” with the transesterification product covalently attached to the catalyst (Chen et al., 2005). However, the regeneration of the active site remained an open question. Daumann et al. studied the reaction in a ${\text{H}}_{2}^{16}$O/H${}_{2}^{18}$O/acetonitrile mixture (Daumann et al., 2012). The observation that ^18^O was incorporated into the hydrolysis product demonstrated the participation of a Zn-OH nucleophile and a reaction pathway analogous to that of alkaline phosphatase seems possible (Figure 9A). The dinucleating macrocycle **HL6** containing an alcohol pendant was designed by Bazzicalupi et al. to model alkaline phosphatase (Bazzicalupi et al., 1999). The dizinc(II) complex contains a Zn-OR and a Zn-OH function and on the basis of ^31^P NMR data and the characterization of the isolated BNPP cleavage product sequential nucelophilic attack by Zn-OR and Zn-OH was proposed (Figure 9B). The complex proved to have a higher reactivity than the parent complex lacking the pendant alcohol group, consistent with Zn-OR presenting the better nucleophile. In contrast to the proposed mechanism for [Zn_2_**HL5**]^2+^, the P-O bond to 4-nitrophenolate is cleaved in the second step which is more in line with its better leaving group property compared to that of the ligand side arm. In other reported model complexes a Zn-bound alcohol group may also adopt the role of an acid catalyst and protonate the leaving group oxygen (Yashiro and Kawahara, 2004).

A DFT study of the cleavage of BDNPP by [Zn_2_**H**_**4**_**L7**(OH_(2)_)]^2+/3+^ revealed a 10.6 kcal mol^−1^ higher energy barrier for alkoxide-mediated attack than for hydroxide-mediated attack (Brown et al., 2016). Liu et al. observed a 93:7 ratio of hydrolysis to ethanolysis product of methyl-(2-chlorophenyl) phosphate in the presence of [Zn_2_**L8**]^4+^ when the reaction was carried out in ethanol containing 3.8 vol% water (Liu et al., 2008a). A detailed analysis taking into account the ionization constant of water in ethanol and the kinetics of the reaction demonstrated that the catalytically active species is [Zn_2_**L8**(μ-OH)]^3+^ and confirmed the large selectivity for activating water as a nucleophile over ethanol. It is also noteworthy that this dizinc(II) complex provides an extremely high rate acceleration of 17 orders of magnitude over the background reaction in 96.2:3.8 ethanol/water (v/v) which is in the same order as the acceleration rates observed for highly efficient enzymatic phosphodiester hydrolysis. The contribution of a synergistic medium effect to this enormous rate enhancement will be discussed in the next section. The catalysis of the methanolysis of a series of methyl aryl phosphate diesters in methanol by the same complex was investigated and the kinetic data were found to be consistent with a two-step mechanism with rate-limiting formation of the phosphorane intermediate following nucleophilic attack of the bridging substrate by a monocoordinate Zn-methoxide (Neverov et al., 2008). Maxwell et al. reported DFT calculations on the [Zn_2_**L8**(μ-OCH_3_)]^3+^-mediated cleavage of 4-nitrophenyl methyl phosphate which gave three viable mechanisms with comparable energy barriers (Maxwell et al., 2013). In all three mechanisms the methoxide dissociates from one Zn(II) and nucleophilic attack on the bridging substrate and expulsion of the leaving group are concerted. The mechanisms differ in whether μ-OCH_3_ acts as the nucleophile or as a general base by deprotonating an external CH_3_OH and in whether leaving group departure is assisted by direct metal-binding or via a metal-bound solvent molecule.

Bosch et al. investigated the role of the second coordination sphere and the influence of hydrogen bonding on substrate binding and catalytic activity (Bosch et al., 2014). The presence of amino and pivaloylamide substituents in ortho position to the pyridine nitrogen in **HL9**–**H**_**2**_**L12** led to lower Michaelis-Menten constants and thus higher catalytic efficiencies for hydrolysing BDNPP compared to the unsubstituted complexes. The orientation of the substituents (symmetric substitution in **HL9** vs. unsymmetric substitution in **HL10**) had a crucial influence on the shape of the rate-pH profile (sigmoidal vs. bell-shaped), the kinetic pK_a_ value, the turnover number, and the maximum reaction rate. The authors also studied the effect of product inhibition and found that at high pH, the dizinc(II) complex of **HL9** formed a less stable product-catalyst complex than [**Zn**_**2**_**L11**], resulting in higher catalytic activity for the former.

For some of the active site mimics that hydrolyzed a DNA model substrate, the DNase activity was also evaluated using plasmid DNA. While there are examples for DNA cleavage activity (Peralta et al., 2010; Anbu et al., 2012; Montagner et al., 2014; Silva et al., 2017; Camargo et al., 2018), it is apparent that factors that are not important for simple phosphodiesters affect the hydrolysis of macromolecular DNA. Binding to a phosphodiester group in DNA can be sterically hindered by a bulky organic ligand (Massoud et al., 2016). On the other hand, metal complexes can show binding preferences for certain nucleotide sequences or structural motifs due to specific ligand-DNA interactions (Camargo et al., 2018). Thus, model studies as those described in this section should not be seen predominantly as a predictive tool for developing efficient DNA cleavage agents, but as a means of studying the role of a dizinc(II) entity in the hydrolysis of the extremely stable phosphodiester linkages that form the backbone of DNA.

### Cleavage of RNA Dinucleotides and RNA Model Substrates

**Figure 10:**
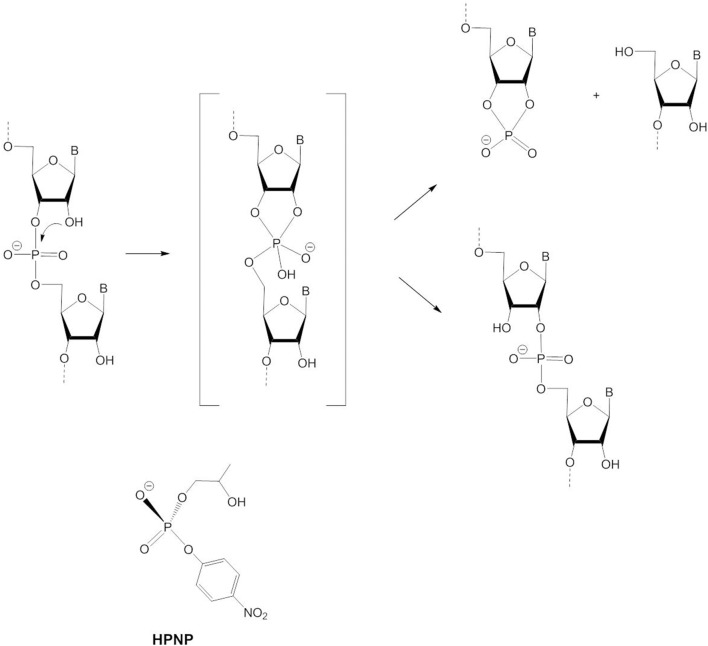
RNA cleavage and 3′ → 2′-isomerization via intramolecular attack by 2′-OH and the RNA model HPNP.

Examples for biological RNA cleavage by a dimetallic site are ribozyme reactions (Steitz and Steitz, 1993) and HIV reverse transcriptase (Davies et al., 1991). RNA is more easily cleaved than DNA due to the 2′-OH group of the ribose ring which can act as an internal nucleophile. As shown in Figure 10, intramolecular attack on the phosphorus leads to the formation of a 2′,3′-cyclophosphate. Thus, RNA is not cleaved by hydrolysis but through transesterification. Whether this reaction proceeds by a stepwise mechanism via a pentacoordinated phosphorane intermediate or by a concerted mechanism via a pentacoordinated transition state has been debated. Evidence is now in favor of a two-step process in the case of the base-catalyzed reaction (Perreault and Anslyn, 1997; Oivanen et al., 1998; Lönnberg et al., 2004). At physiological pH the pentacoordinate phosphorane is monoanionic and relatively stable so that it can undergo pseudorotation. As a consequence, migration of the phosphodiester group to the 2′-position of the ribose ring can compete with RNA cleavage (Figure 10). In line with the principle of microscopic reversibility the leaving group has to depart from an axial position as the nucleophile attacks at an axial position. Under alkaline conditions the dianionic phosphorane is too short-lived and pseudorotation to an intermediate with the 3′-oxygen and a negatively charged oxygen in the axial positions is too energetically unfavorable for 3′ → 2′ isomerization to occur. Experimental and computational data suggest that the reaction switches to a concerted pathway involving a dianionic pentacoordinate transition state, when the transesterification to the cyclophosphate is catalyzed by metal ions (Bunn et al., 2007; Humphry et al., 2008; Tsang et al., 2009; Edwards et al., 2010). Isomerization is not possible in this case.

2-Hydroxypropyl-*p*-nitrophenyl phosphate (HPNP, Figure 10) is a popular model for the phosphodiester linkages in RNA. The enhanced catalytic activity of various dinuclear zinc(II) complexes relative to their mononuclear analogs is usually attributable to double Lewis acid activation of HPNP adopting a bridging coordination mode. Like the catalysis of the hydrolysis of DNA models, HPNP transesterification is often more efficiently catalyzed by dizinc(II) complexes with unsymmetric ligands that have more available coordination sites to bind the substrate and water/hydroxide for base catalysis (Carlsson et al., 2004; Jarenmark et al., 2008). As the 2′-OH group is an internal, thus more efficient nucleophile, Zn-OH does not participate in the reaction mechanism as a nucleophile but serves as a base catalyst. Depending on the model complex and the solvent system, different conclusions were reached regarding the question of whether Zn-OH acts as a general or a specific base catalyst. In general base catalysis, deprotonation of the 2′-OH group by Zn-OH occurs concurrently with nucleophilic attack, while in specific base catalysis the 2'-oxyanion is formed in a pre-equilibrium step prior to rate-determining substrate cleavage.

The dinuclear Zn(II) complex of **L8** is one of the most efficient RNA/HPNP cleavage catalysts reported to date. In methanol, in the presence of one equivalent CH_3_O^−^ [Zn_2_**L8**]^4+^ gives a 10^8^-fold rate acceleration of the cleavage of HPNP over the methoxide-catalyzed reaction (Neverov et al., 2006). Tsang et al. carried out a kinetic analysis of the transesterification of different 2-hydroxypropyl-aryl and alkyl esters by [Zn_2_**L8**(OCH_3_)]^3+^ and found that the reaction proceeds through a transition state in which the departure of the leaving group has progressed to 45% (Tsang et al., 2009). A DFT study by Maxwell et al. revealed three plausible, competing mechanisms, all involving bridging substrate coordination (Maxwell et al., 2013): (i) direct nucleophilic attack by the metal-bound HPNP alkoxide concurrent with the cleavage of the leaving group bond—the departure of the leaving group is assisted by a terminally bound methanol acting as an H bond donor; (ii) rate-limiting nucleophilic attack through a general base mechanism leading to a phosphorane intermediate—subsequent bond cleavage is assisted by metal binding and (iii) nucleophilic attack through a general base mechanism and leaving group departure occurring in concert—the expulsion of the leaving group is assisted by hydrogen bonding with a terminally coordinated methanol. While experimental data were reported for the [Zn_2_**L8**(OCH_3_)]^3+^-promoted transesterification of HPNP and 2-hydroxypropyl-phenyl phosphate that are consistent with both a concerted and a stepwise mechanism, it has been argued that a stepwise pathway may be more likely because a strong electrostatic interaction between the highly charged dizinc(II) site and the putative dianionic phosphorane should stabilize the intermediate and the transition state leading to it (Bunn et al., 2007). Energetics calculations indicated that the transition state of the catalyzed reaction is stabilized by about −21 to −23 kcal mol^−1^ relative to the transition state of the methoxide reaction. The charge of a phosphodiester increases from −1 to −2 when the catalyst-substrate complex proceeds to the transition state. It has been predicted that the coordination of two metal ions to a phosphate ester monoanion has the same effect as neutralizing it. It is believed that substrate binding to [Zn_2_**L8**(OCH_3_)]^3+^ in alcoholic medium takes place in two steps (Bunn et al., 2007); The substrate binds initially as a monodentate ligand to one Zn^2+^ ion and then rearranges to the catalytically active species with a bridging coordination mode allowing double Lewis acid activation. For substrates with a good leaving group such as 4-nitrophenolate this rearrangement is rate-determining and the following steps of the transesterification reaction are fast. In the case of substrates with a poor leaving group complete equilibrium binding of the substrate occurs and the rate determining step is a chemical one that depends on the pK_a_ value of the leaving group. Nucleophilic attack is rate-determining when the pK_a_ of the leaving group is lower than that of the nucleophile. When the leaving group pK_a_ is greater, fission of the leaving group bond becomes rate-limiting. The change of the rate-determining step from formation to breakdown of the phosphorane intermediate manifests itself as a break in the Brønsted plot (plot of logk_cat_ vs. leaving group pK_a_) at the point where the effective pK_a_ of the leaving group and the nucleophile are the same. For the transesterification in ethanol in the presence of [Zn_2_**L8**(OC_2_H_5_)]^3+^, general-base catalyzed deprotonation of the 2′-OH group by Zn-OC_2_H_5_ was proposed. Specific-base catalysis by an external ethoxide could be excluded, because the cleavage rate in ethanol exceeded the diffusion limit (Liu et al., 2008b). Support for concerted nucleophilic attack and loss of the leaving group comes from a study of the reaction of [Zn_2_**L8**(OR)]^3+^ with a stable phosphonate analog of HPNP (Edwards et al., 2010). If the slow cleavage of 2-hydroxypropyl phenyl phosphonate were to proceed via a five-coordinate phosphorane intermediate, isomerization to 1-hydroxypropyl phenyl phosphonate should be observed, which was not the case.

**Figure 11:**
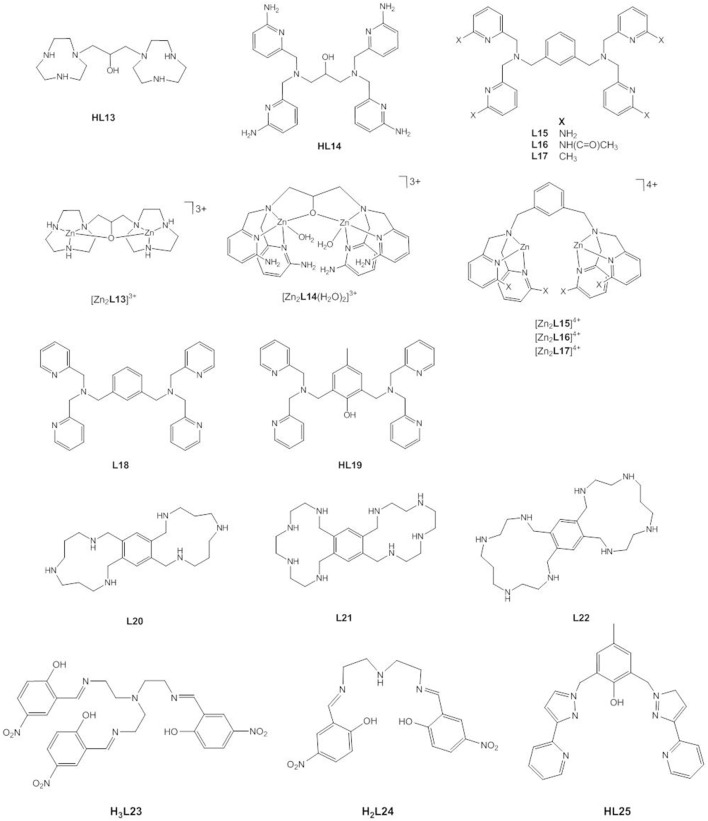
Chemical structures of ligands **HL13**–**HL25** and the dinuclear Zn(II) complexes [Zn_2_**L13**]^3+^, [Zn_2_**L14**(H_2_O)_2_]^3+^, [Zn_2_**L15**]^4+^, [Zn_2_**L16**]^4+^ and [Zn_2_**L17**]^4+^.

In contrast to the general-base catalyzed cleavage of HPNP by [Zn_2_**L8**(OC_2_H_5_)]^3+^ in ethanol, experimental data for the related dizinc(II) complex [Zn_2_**L13**(OH_2_)]^3+^ (Figure 11) have been interpreted in terms of both general and specific base catalysis. Concerted nucleophilic attack and leaving group loss with specific-base catalysis in aqueous solution is now favored (Iranzo et al., 2003b; Yang et al., 2005; Humphry et al., 2008). Likewise, two conflicting computational studies were reported that came to different conclusions. DFT calculations that were most consistent with the experimental data found the substrate to bind via the two phosphoryl oxygens in a bridging mode and via the nucleophilic 2′-OH group. The pre-equilibrium step involving the activation of the 2′-OH group through specific-base catalysis by Zn-OH is followed by the concerted nucleophilic attack and cleavage of the leaving group bond (Gao et al., 2011). Similar to the mechanism (i) in the DFT study of [Zn_2_**L8**]^4+^, [Zn_2_**L13**]^3+^ alters the loose transition state of the uncatalyzed reaction to a more associative or tight one. The second theoretical study published earlier found the same substrate binding mode but proposed a two-step pathway with general base catalysis (Fan and Gao, 2010). It was pointed out that the large rate accelerations of the cleavage of RNA models provided by [Zn_2_**L13**]^3+^ were due to the dominant role of electrostatics in stabilizing the dianionic transition state (Iranzo et al., 2003b; Yang et al., 2005, 2007). The densely charged core of two close packed Zn^2+^ ions binds the transition state with high affinity, leading to a transition state stabilization that is ca. 50% of that estimated for the corresponding enzymatic reaction. Kinetic analysis revealed 2.1 kcal mol^−1^ of greater stabilization of the transition state for the cleavage of uridylyl(3′ → 5′)uridine (UpU) compared to the transition state for the cleavage of uridine-3′-4-nitrophenyl phosphate (UpPNP) which demonstrates that the transition state stabilization of the developing negative charge on the leaving group oxygen of UpU is stronger than the stabilizing interaction between the catalyst and the C-2′ oxyanion nucleophile at the rate-determining transition state of UpPNP cleavage (O'Donoghue et al., 2006).

Mikkola, Williams and coworkers studied the hydrolysis of HPNP, UpU, and uridine-3′-alkyl phosphates by [Zn_2_**L14**(H_2_O)_2_]^3+^ and observed that the complex not only provides an enormous 10^6^-fold rate acceleration of the cleavage reaction in aqueous solution, but also catalyzes the isomerization to the corresponding uridine-2′-alkyl phosphates (Feng et al., 2006; Linjalahti et al., 2008; Korhonen et al., 2012). This means that the dizinc(II) entity stabilizes the phosphorane intermediate sufficiently to allow pseudorotation, and is clear evidence for a stepwise mechanism. It was proposed that the expulsion of the leaving group is the rate-determining step and is general-acid catalyzed. Cocrystallization of the zinc(II) complex with 4-nitrophenyl phosphate confirmed that the phosphoryl oxygen atoms of the bridging phosphate ester are in hydrogen bonding distance of the four amino substituents. By serving as second-sphere H-bond donors, the amino groups contribute to the stabilization of the dianionic phosphorane and provide a further 10^3^-fold rate enhancement of the cleavage of HPNP compared to the unsubstituted complex due to tighter binding of the substrate to the catalyst and to the transition state. Again, it becomes clear that charge neutralization by an electrophilic catalyst plays a dominant role. The dinuclear complex stabilizes the phosphorane to the same extent as complete neutralization of one negative charge and to an extent that enables 3′ → 2′ isomerization. The isomerization is catalyzed less efficiently than the cleavage reaction. While binding to the zinc(II) complex stabilizes the phosphorane, it restrains its conformational change required for isomerization to occur.

Interestingly, Mohamed and Brown found that the dizinc(II) complexes of **L15**, **L16**, and **L17**—having amino, acetamido and methyl substituents, respectively—gave similar increases in k_cat_ for the cleavage of HPNP in methanol (Mohamed and Brown, 2010). The kinetic data were interpreted to suggest that hydrogen bonding effects are important for catalysis, but less so for substrate binding. The key conclusion, however, was that the creation of a hydrophobic pocket by the methyl substituents is just as effective as hydrogen bonding. By contrast, methylation of the coordinating nitrogens in **L8** reduces the catalytic efficiency and the synergism between the two Zn^2+^ ions, most likely due to steric effects that impair substrate binding (Song et al., 2012).

Besides introducing substituents, the linker between the two triaza macrocycles in **L8** was varied (Liu et al., 2009; Guo et al., 2011). When more rigid aromatic linkers were employed, the synergistic effect of the two metals varied between 5- and 700-fold (Guo et al., 2011). Replacing the propylene linker in **L8** with a butylene linker led to an increase in the activation energy ΔG_cat_ of around 1–1.6 kcal mol^−1^, which was attributed to a less tightly bound substrate-catalyst complex at the transition state (Liu et al., 2009). The presence of the 2-propoxy linker in [Zn_2_**L13**]^3+^ leads to a 37,000-fold decrease in the catalytic activity toward HPNP in methanol compared to [Zn_2_**L8**(OCH_3_)]^3+^ (Mohamed et al., 2009). Possible reasons for this include the reduction in Lewis acidity of the Zn^2+^ ions, the higher coordination number of the Zn^2+^ ions, decreased stabilization of the negative charge development in the transition state and the loss of conformational flexibility (Mohamed et al., 2009; Maxwell et al., 2013). DFT calculations showed that the Zn(II)^…^Zn(II) distance in [Zn_2_**L8**(OCH_3_)]^3+^ expands from ca. 3.6 Å to over 5 Å in the intermediates and transition states (Maxwell et al., 2013). Likewise, the dinuclear Zn(II) complex of **L18** is more active in methanol than the analogous complex of **HL19** (Mohamed et al., 2009). Energetics calculations showed a greater stabilization of 3.7 kcal mol^−1^ of the transition state by the former compared to the latter. Interestingly, the situation seems to be different in aqueous solution. In water, the bridging linker is believed to be crucial to achieve cooperativity between the metal ions (Iranzo et al., 2003a; Morrow, 2008). There is no doubt about the importance of medium effects. While the zinc(II) complex of **HL13** is an efficient catalyst in aqueous solution, in ethanol it accelerates the transesterification of HPNP by an impressive 12 orders of magnitude relative to the background reaction at the same ${}_{s}^{s}pH$ (Bunn et al., 2007). It has been proposed that the reduced polarity of the solvent results in desolvation of the ionic components and a better solvation and stabilization of the charge-dispersed transition state (Bunn et al., 2007; Korhonen et al., 2012). The effect of a lower dielectric constant on the binding of ions of opposite charge will increase the catalyst-substrate binding constant. Energetics calculations gave a $\Delta G_{\text{stab}}^{\neq }$ of −21 kcal mol^−1^ for the [Zn_2_**L8**]^4+^-mediated cleavage of HPNP in methanol which is close to the $\Delta G_{\text{stab}}^{\neq }$ expected for highly efficient phosphodiesterase enzymes (Bunn et al., 2007).

For [Zn_2_**L19**(μ-OH)]^2+^, a medium effect on the reaction pathway was also described (Selmeczi et al., 2007). DFT calculations indicated that the hydroxypropyl arm of the bridging HPNP is oriented at the hydrogen bonding distance to the μ-OH group. This H-bond facilitates the deprotonation of the attacking nucleophile by the hydroxido bridge. In aqueous solution, a further proton transfer to an external hydroxide takes place, while in a non-aqueous medium (DMSO), the protonated μ-OH_2_ shifts to a terminal position. In both cases DFT calculations agreed with the concurrent deprotonation of 2′-OH and P-O bond formation, leading to a pentacoordinate phosphorane which, however, appears to be not as viable in the non-aqueous medium. In DMSO, the μ_2_-κ^1^*O*:κ^1^*O*′-bridging coordination mode of the cyclophosphate product is in equilibrium with the cyclophosphate forming a monoatomic bridge. This “phosphate shift” was not observed in aqueous solution.

Bim et al. studied dinuclear Zn(II) complexes with the conformationally constrained bis-polyazamacrocycles **L20** – **L22** (Bím et al., 2016). Only [Zn_2_**L20**]^2+^ showed catalytic activity in aqueous buffer. Kinetic data and DFT calculations were consistent with two mechanistic scenarios with similar energy barriers and with the substrate coordinating via the two phosphoryl oxygens to both Zn and via the deprotonated 2-hydroxy group to one Zn (Zn1). In mechanism (1) nucleophilic attack and dissociation of the leaving group take place in two steps. In (2) an additional water molecule binds to Zn2 and the mechanism becomes a one-step process. By contrast, DFT calculations for the unsymmetric complex [Zn_2_**L4**(μ-OH)(OH)] clearly favor a concerted associative mechanism for HPNP transesterification (Daver et al., 2016). While the deprotonation of the 2-OH nucleophile in a pre-equilibrium step was proposed on the basis of experimental data (Jarenmark et al., 2010), the DFT calculations indicated a significantly lower energy barrier for a general-base mechanism in which the deprotonation of the bridging HPNP by Zn-OH and nucleophilic attack occur concomitantly.

Three-metal cooperativity was recently reported for the trinuclear complex [Zn_3_(**L23**)_2_(H_2_O)_4_]^.^H_2_O^.^2DMF (Joshi et al., 2018). It was suggested that the cooperative action of the three metals comprising double Lewis acid activation of the bridging HPNP and base catalysis by the third Zn^2+^ ion is assisted by the cup-shaped cavity of the complex. The trinuclear complex gives a ca. 4-fold higher k_cat_ value than the analogous dinuclear complex [Zn_2_(**L24**)_2_(H_2_O)_2_](ClO_4_)_2_, for which monodentate substrate coordination to Zn1 and base catalysis by Zn2-OH were proposed.

As discussed in a previous section, there are conflicting data in the literature on the correlation between the catalytic activity of phosphoesterase models and the Lewis acidity of the metal ion(s). Arora et al. compared the rate acceleration of HPNP transesterification provided by [Zn_2_**L25**(H_2_O)_x_(OH)_y_]^n+^ and the analogous Co(II) and Mn(II) complexes and found a linear correlation of the rate constant k_2_ with the Z_eff_/r value of the metal ion (Arora et al., 2012). Thus, Lewis acid activation of the phosphorus is more important than activation of the nucleophile in this case. It may be relevant that the nucleophile is an internal one that is *per se* more efficient than the external one for general phosphate ester substrates.

As is evident from the above, in the majority of studies the model substrate HPNP was used. Some caution must be exercised when applying conclusions drawn from these analyses to RNA. It has been pointed out in the literature that the 2-OH group in HPNP is more flexible than the ribose 2′-OH and also has a higher pK_a_ value (Korhonen et al., 2012). Hydrophobic and π-stacking interactions between the linker moiety or heteroaromatic binding site of a dinuclear ligand and the 4-nitrophenyl group have been demonstrated to enhance substrate binding and to increase the catalytic activity (Bazzicalupi et al., 2004). Leivers and Breslow showed that this can incorrectly suggest cooperativity between two metal centers (Leivers and Breslow, 2001). Furthermore, the literature shows that the rate-determining step depends on the nature of the leaving group, when the reaction proceeds through the A_N_+D_N_ mechanism (vide supra). Mikkola and coworkers published a comprehensive analysis of the dizinc(II) complex-mediated cleavage of uridine-3′-aryl and uridine-3′-alkyl phosphates. The observed cooperativity of the two metals in dinuclear catalysts changes with the acidity of the leaving group of the substrate. In the case of alkyl groups, the cooperativity decreases with decreasing acidity, whereas in the case of aryl phosphates the cooperativity increases with decreasing acidity—i.e., as nucleophilic attack becomes more rate-determining. They concluded that there is no universal mechanism for the transesterification of RNA and its analogs that covers all substrate-catalyst combinations (Korhonen et al., 2013).

### Phosphodiester Hydrolysis Catalyzed by Heterodinuclear Fe(III)Zn(II) Complexes

**Figure 12:**
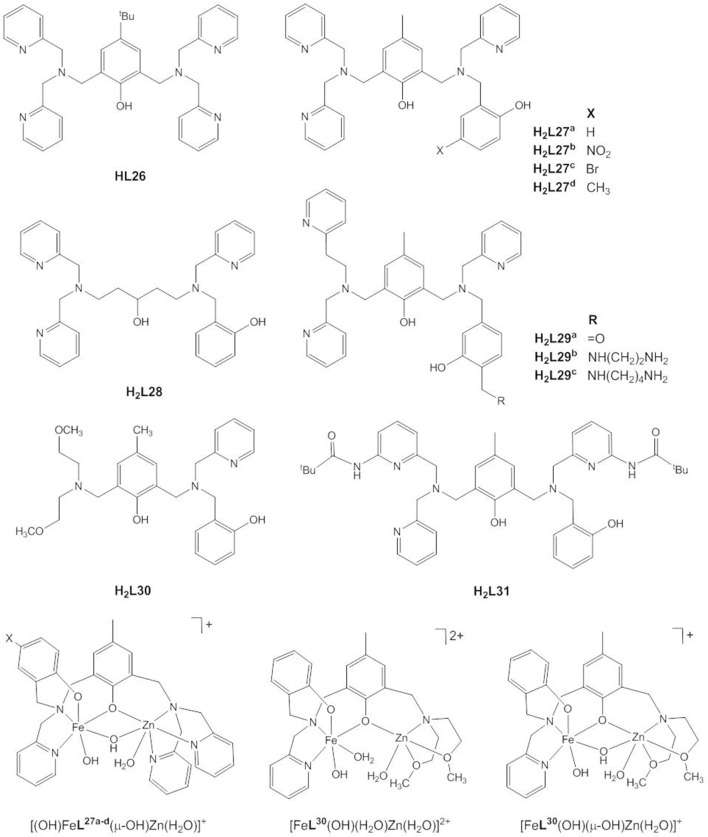
Chemical structures of ligands **HL26** - **H**_**2**_**L31** and the catalytically active species [(OH)Fe**L27**^**a−d**^(μ-OH)Zn(H_2_O)]^+^, [Fe**L30**(OH)(H_2_O)Zn(H_2_O)]^2+^, and [Fe**L30**(OH)(μ-OH)Zn(H_2_O)]^+^.

Following earlier work by Borovik and Que and by Wieghardt and coworkers who synthesized heterodinuclear, carboxylate-/hydroxide-bridged Fe(III)/M(II) complexes to model iron-oxido proteins (Borovik et al., 1988; Hotzelmann et al., 1992), a number of biomimetic studies were targeted specifically at the mechanism of the heterodinuclear Fe(III)Zn(II) site of plant PAPs. Pathak et al. reported the Fe(III)Zn(II) complex of the symmetric ligand **HL26** (Pathak et al., 2018), but usually unsymmetric ligands with the two binding sites differing in the number and/or nature of the donor atoms are employed to stabilize the heterodimetallic site (Figure 12). **H**_**2**_**L27**^**a**^ was specifically designed to provide a hard *N*_2_*O*_4_ site for the trivalent Fe(III), and a softer *N*_3_*O*_3_ site for the divalent Zn(II) in the presence of additional bridging carboxylate or hydroxido ligands and to model the terminal tyrosinate ligand in PAP (Lanznaster et al., 2002; Neves et al., 2007). Single crystal structures of both the acetate- and hydroxido-bridged complexes, [Fe(III)Zn(II)**L27**^**a**^(μ-CH_3_COO_2_)_2_]ClO_4_ and [(H_2_O)Fe(III)Zn(II)**L27**^**a**^(μ-OH)](ClO_4_)_2_, could be obtained showing that the M(III)^…^M(II) distance decreases from 3.490(9) to 3.040(1) Å when the carboxylate ligands are replaced with a μ-OH ligand. The Fe(III)^…^Zn(II) distance in the latter is slightly shorter but comparable to that of 3.20 Å in red kidney bean PAP (Klabunde et al., 1996).

While in the majority of the Fe(III)Zn(II) complexes of **HL26**–**H**_**2**_**L31** the metals are bridged by two acetates in the solid state, in solution dissociation of the acetate ligands leads to [(H_2_O)Fe**L**(μ-OH_2_)Zn(H_2_O)]^n+^, [(H_2_O)Fe**L**(μ-OH)Zn(H_2_O)]^(n−1)+^, [(OH)Fe**L**(μ-OH)Zn(H_2_O)]^(n−2)+^, and [(OH)Fe**L**(μ-OH)Zn(OH)]^(n−3)+^ species, depending on the pH value (Lanznaster et al., 2002; Neves et al., 2007; Peralta et al., 2010; Piovezan et al., 2010; Jarenmark et al., 2011; Roberts et al., 2015; Pathak et al., 2017). Rate-pH profiles and potentiometric titration data indicate that [(OH)Fe**L**(μ-OH)Zn(H_2_O)]^n+^, which is present in weakly acidic solution is the catalytically active species. In all cases the kinetic data are consistent with the mechanism of PAP proposed by Klabunde et al. (Figure 2B, Klabunde et al., 1996). The phosphodiester replaces the Zn-bound water in a monodentate binding mode while Fe(III)-OH acts as the nucleophile.

The effect of substituents in para position to the terminally-bound phenolate oxygen in **H**_**2**_**L27**^**a−d**^ confirms the role of Fe(III) as the provider of the nucleophile. Electron-withdrawing groups (NO_2_, Br) lead to a decrease in the hydrolysis rate, while electron-donating groups (CH_3_) enhance the phosphodiesterase activity (Peralta et al., 2010). The higher the electron-donating property of the ligand, the lower the Lewis acidity of the metal ion is and the weaker the M-OH interaction is. When there is less pull of the electron density by the metal, the metal-bound hydroxide presents a stronger nucleophile. The observation that the analogous Ga(III)Zn(II) complex of **H**_**2**_**L27**^**a**^ hydrolyses BDNPP more efficiently than does the Fe(III)Zn(II) complex is also in line with nucleophilic attack by M(III)-OH (Smith et al., 2007). The authors of the study attributed the higher catalytic activity of the Ga(III)Zn(II) complex to the importance of the higher lability of Ga(III) compared to Fe(III) when product release is the rate-determining step. However, the difference in the pK_a_ value of Ga(III)-OH_2_ (pK_a_ = 5.59) and Fe(III) (pK_a_ = 4.86) may also suggest that Ga(III) provides a stronger nucleophile.

Ferreira et al. carried out DFT calculations on the reaction mechanism of the hydrolysis of dimethyl phosphate by the closely related complex [(OH)Fe**L28**(μ-OH)Zn]^+^ (Ferreira et al., 2008). The optimized structure of the substrate-catalyst complex showed that substrate binding is stabilized by a H-bond between Fe-OH and a phosphoryl oxygen with a Gibbs free energy variation of −55.1 kcal mol^−1^. The hydrolysis reaction proceeds by a two-step associative mechanism. The first, rate-determining step involves the nucleophilic attack of Fe-OH at the Zn-bound phosphodiester resulting in the pentacoordinate phosphorane intermediate. The movement of the OH group toward the phosphorus and P-O bond formation is accompanied by a fast proton transfer from OH to the phosphoryl oxygen. In the second step, the simultaneous proton transfer from P-OH to the leaving group oxygen and breaking of the leaving group bond lead to the release of CH_3_OH.

**H**_**2**_**L29**^**a**^, **H**_**2**_**L29**^**b**^, and **H**_**2**_**L29**^**c**^ were synthesized to model secondary interactions between the phosphate ester substrate and positively charged amino acid residues in the active site of PAP (Silva et al., 2017). The presence of the side chains in **H**_**2**_**L29**^**b**^ and **H**_**2**_**L29**^**c**^ led to a decrease in the pK_a_ value of Fe(III)-OH_2_ by 0.6 and 0.8 pH units compared to the parent complex and to a shift of the redox potential of Fe(III) to less negative values. This was rationalized by hydrogen bonding between the ammonium group of the side chain and the bridging hydroxide as observed in the optimized solid-state structures of [Fe**L29**^**b**^(OH)(μ-OH)Zn(H_2_O)](ClO_4_)_2_ and [Fe**L29**^**c**^(OH)(μ-OH)Zn(H_2_O)](ClO_4_)_2_. The higher k_cat_ and lower K_m_ values of [Fe**L29**^**b**^(OH)(μ-OH)Zn(H_2_O)](ClO_4_)_2_ and [Fe**L29**^**c**^(OH)(μ-OH)Zn(H_2_O)](ClO_4_)_2_ compared to [Fe**L29**^**a**^(OH)(μ-OH)Zn(H_2_O)](ClO_4_)_2_ reflect the enhanced binding affinity of the substrate for the side-chain bearing complexes. The changes in K_m_ were found to correlate with the proximity of the side chain to the phosphate group in the optimized structures of the catalyst-substrate complexes. Camargo et al. attached one and two pyrene moieties via a diamine spacer to the ortho position of the phenol ring in **H**_**2**_**L27**^**a**^ (Camargo et al., 2018). They suggested that the 6-fold increase in K_ass_ for BDNPP was due to H-bond formation and hydrophobic interactions between pyrene and 4-nitrophenol. The determination of the activation parameters for BDNPP hydrolysis revealed a decrease of Δ*H*^≠^ by ca 10 kJ mol^−1^ with respect to the corresponding complex having a carbonyl group as a substituent, and this was attributed to hydrogen bonding and the stabilization of the negatively charged transition state. However, this favorable enthalpic contribution was offset by a less favorable Δ*S*^≠^, probably due to a higher degree of structural organization in the transition state.

The unsymmetric ligand **H**_**2**_**L30** provides an *N*_2_*O*_2_ and an *NO*_3_ site. The speciation plot and rate-pH profile suggest that its Fe(III)Zn(II) complex mimics the mechanistic flexibility of PAP (Roberts et al., 2015). At a low pH, [Fe**L30**(OH)(H_2_O)Zn(H_2_O)]^2+^ is the active species and the terminally bound OH acts as the nucleophile. At higher pH, the bridging OH of [Fe**L30**(OH)(μ-OH)Zn(H_2_O)]^+^ becomes the nucleophile. [Fe**L30**(OH)(μ-OH)Zn(H_2_O)]^+^ is the better catalyst. Jarenmark et al. synthesized [FeZn**L4**(μ-CH_3_COO)_2_(CH_3_OH)]PF_6_ to model the distinct donor atoms as well as the different coordination numbers of Fe(III) and Zn(II) in PAP (Jarenmark et al., 2011). The complex hydrolyzes BDNPP and also shows some activity toward HPNP. At high pH the complex converts to an inactive μ-oxido-bridged dimer of heterodinuclear dimers.

**HL9**, **HL10**, and **H**_**2**_**L31** contain sterically demanding pivaloyl-amide substituents. A detailed kinetic analysis of the hydrolysis of BDNPP by the respective heterodinuclear Ga(III)Zn(II), homodinuclear Zn(II)_2_, mononuclear Zn(II), and Ga(III) complexes gave insight into the influence of the secondary coordination sphere, the effect of the properties of the binding site and the role of the heterodinuclear site (Bosch et al., 2016). Hydrogen bonding capacity shifts the pH optimum to higher pH values. The presence of H-bond donating substituents also leads to higher hydrolysis rates and higher catalytic efficiencies, especially when the two H-bond donors are located proximal to the Zn(II) site. The Ga(III)Zn(II) complex of **HL9** hydrolyses BDNPP faster and with larger turnover numbers than the corresponding **HL10** complex. However, the introduction of the H-bond donating substituents decreases the substrate affinity, which may be a steric effect. A comparison of **HL9** and **H**_**2**_**L31** showed that the pH optimum shifts to lower pH values when the binding site becomes more electron-rich. The catalytic activity of the heterodinuclear complex of **HL10** is greater than the sum of the activities of the mononuclear Zn(II) and Ga(III) complexes confirming the cooperativity of the metals in the dimetallic site. The Ga(III)Zn(II) complex of **HL9** hydrolyzes BDNPP about 20 times faster at pH 7 than the dizinc(II) complex. Interestingly, K_m_ is three times higher for the Ga(III)Zn(II) complex. A weaker substrate affinity of the heterodinuclear complex compared to the corresponding homodinuclear Zn(II)_2_ complex was also seen for [Fe(III)Zn(II)**L26**(μ-CH_3_COO)_2_]^2+^(Pathak et al., 2018). It appears that the electronic effect of the heterodimetallic site on substrate and/or nucleophile activation is more important than the formation of the catalyst/substrate complex.

### Hydrolysis of Phosphotriesters and Organophosphates

**Figure 13:**
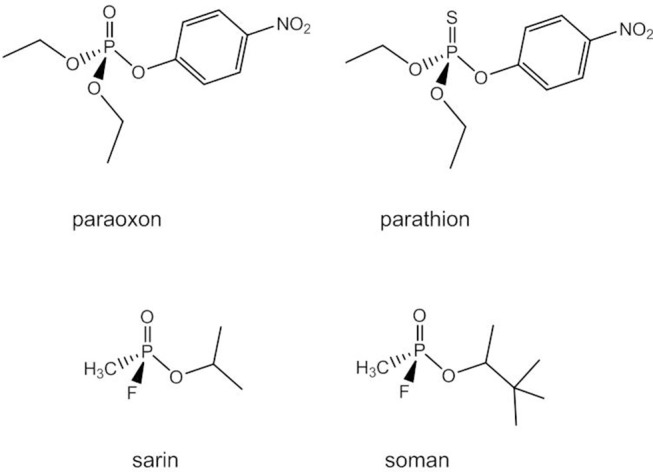
Synthetic phosphotriesters and organophosphates.

Due to their neutral charge phosphotriesters are more easily hydrolyzed at pH 7 than phosphodi- and -monoesters. The mechanism of the uncatalyzed reaction is believed to be associative with both a two-step addition-elimination and a concerted pathway being possible. Phosphotriesters do not occur naturally. Synthetic organophosphate triesters have been widely used as pesticides and insecticides (e.g., paraoxon, parathion, Figure 13). In mammals, they cause nerve and organ failure due to their ability to inhibit acetylcholinesterase and some highly toxic organophosphorus compounds such as sarin and soman are employed as chemical warfare agents (Raushel, 2002). Bacterial phosphotriesterases can degrade phosphotriesters and their analogs into less toxic diesters and have probably evolved in response to the intense application of synthetic organophosphates in agriculture (Donarski et al., 1989; Dumas et al., 1990).

**Figure 14:**
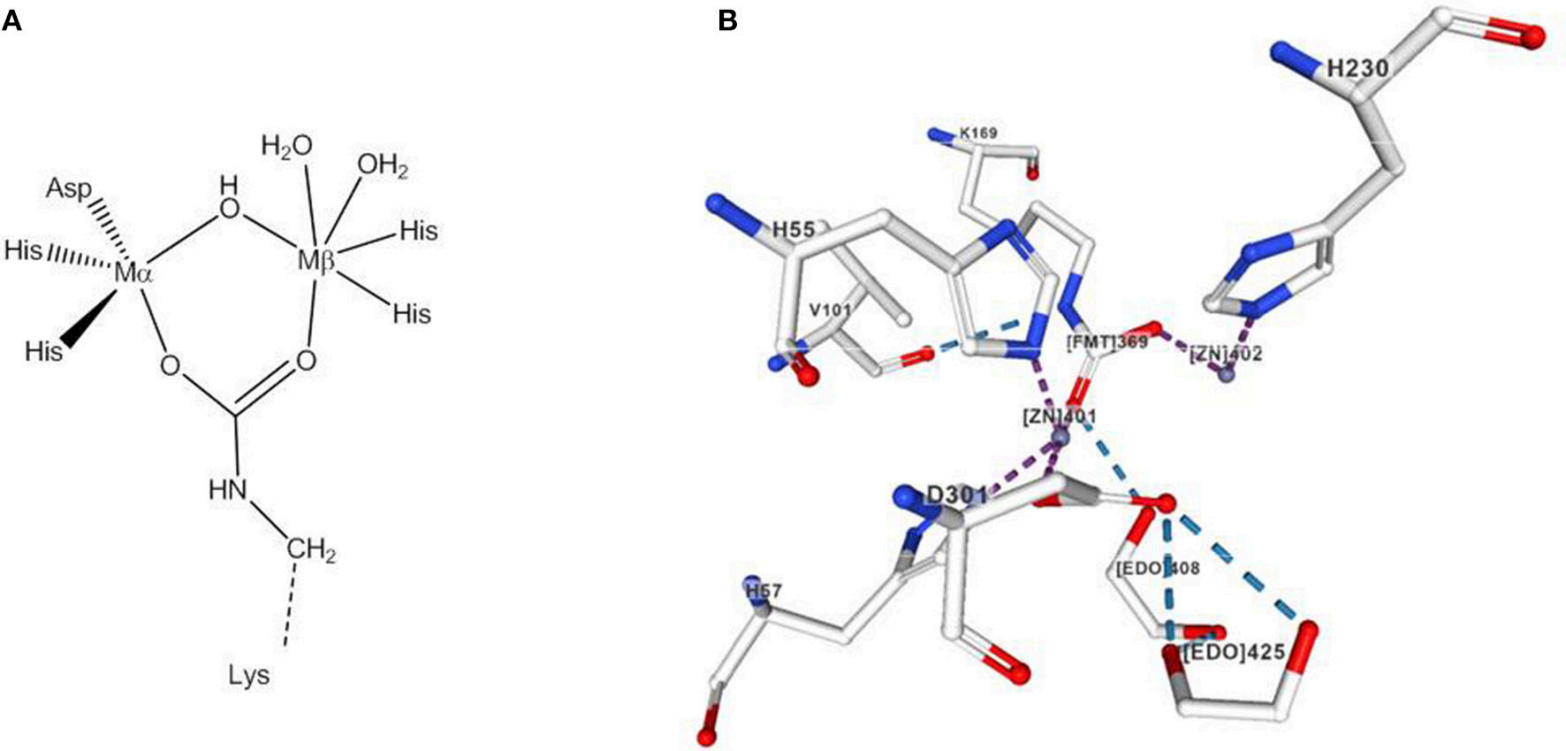
**(A)** Active site of OpdA and OPH. **(B)** View of the active site region of the X-ray structure of phosphotriesterase isolated from *Pseudomonas diminuta* created with the PDB 1HZY and associated publication (Benning et al., 2001). NGL Viewer (Rose et al., 2018) and RCSB PDB.

Two of the best-studied phosphotriesterases are the Zn-containing organophosphate degrading enzymes from *Agrobacterium radiobacter* (OpdA) and organophosphate hydrolase from *Pseudomonas diminuta* (OPH). Glycerophosphodiesterase from *Enterobacter aerogenes* (GpdQ) is also known to hydrolyze organophosphate triesters. OpdA and OPH share a high sequence and structure homology (Yang et al., 2003). In their active site two Zn^2+^ ions, referred to as α- and β-site are bridged by a carboxylated lysine side chain and a hydroxide/water (Benning et al., 2001; Figure 14). Small-molecule models of OpdA have been previously reviewed (Daumann et al., 2014).

The mechanism of enzymatic phosphotriester hydrolysis was investigated in theoretical studies that indicated an associative pathway involving binding of the phosphoryl oxygen to the β-site and nucleophilic attack by the bridging hydroxide (Ely et al., 2010, 2011). On substrate coordination, the μ-OH bond to the β-site weakens and the bridging hydroxide shifts to a *pseudo*-terminal position. However, like PAPs, phosphotriesterases appear to exhibit mechanistic flexibility. Experimental and theoretical studies showed that in the case of the Co(II) form of OpdA under alkaline conditions, the μ-OH group shifts to the β-site following substrate binding and a hydroxide or water from the environment coordinates terminally to the α-site to act as the reaction-initiating nucleophile (Ely et al., 2010, 2011). There is also evidence that the rate-determining step varies with the nature of the leaving group. For leaving groups with a pK_a_ > 7, P-O bond cleavage seems to be rate-determining (Ely et al., 2010). Based on kinetic and crystallographic data and computational modeling of the Fe(II)Zn(II) form of OpdA Jackson et al. proposed that the bridging hydroxide serves as a base and deprotonates a water molecule terminally coordinated to the α-site (Jackson et al., 2008). However, such a mechanism appears unlikely for the dizinc(II) form of OPH (Kim et al., 2008).

**Figure 15:**
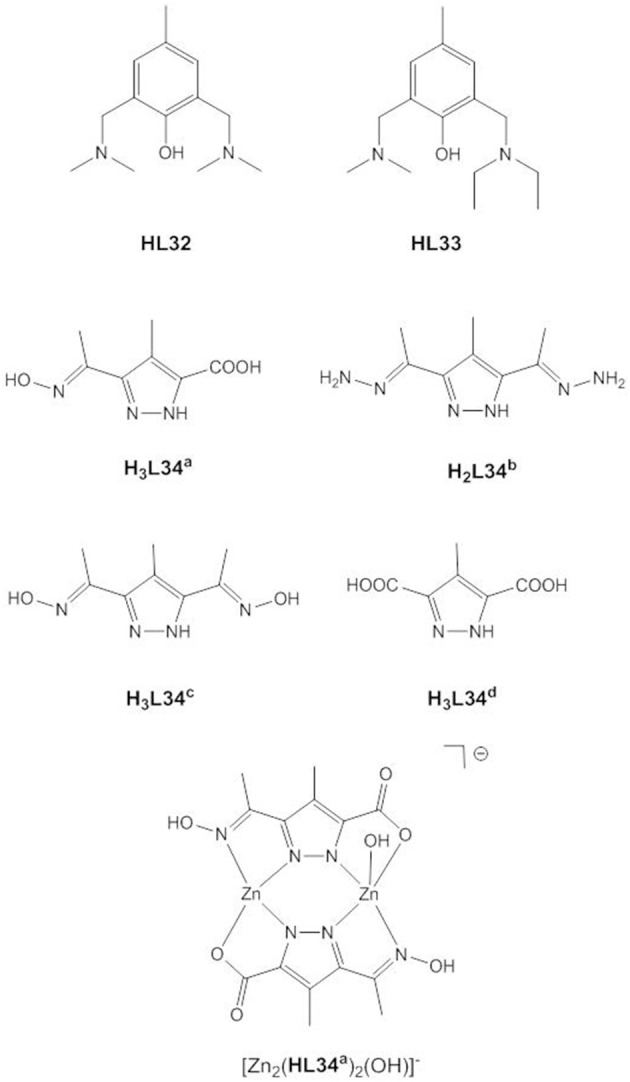
Chemical structures of ligands **HL32** - **H**_**2**_**L34** and [Zn_2_(**HL34**^**a**^)_2_(OH)]^−^.

To address the need for effective bioremediators to decontaminate organophosphate-containing water and soil, biomimetic zinc(II) sites have been assembled into metal-organic frameworks or onto graphene oxide, and promising catalysts are described in the recent literature (Jacques et al., 2008; Ma et al., 2017; Xia et al., 2017). However, detailed mechanistic studies using model complexes and phosphotriesters are rarely reported and most of our current mechanistic understanding of dizinc(II) phosphotriesterases stems from theoretical studies such as those described above. In contrast to the polymer and metal organic framework-based active catalysts, little success has been achieved so far in the development of low-molecular-weight dizinc(II) phosphotriesterase mimics. Only modest phosphotriesterase activity was observed for the small number of dinuclear zinc(II) complexes investigated (Figure 15). The low activities of [Zn_2_**L32**(μ-CH_3_COO)(CH_3_COO)_2_(H_2_O)] (Tamilselvi and Mugesh, 2010) and [Zn_2_**L33**(μ-CH_3_COO)(CH_3_COO)_2_(H_2_O)] (Umayal and Mugesh, 2011) toward 4-nitrophenyl diphenyl phosphate was attributed to inhibition by the phosphodiester hydrolysis product that binds in a bridging mode to the dizinc(II) site. Two of the few examples of dizinc(II) complexes with phosphotriesterase activity were reported by Guo et al. (Guo et al., 2015). [Zn_2_**L5**]^+^ and [Zn_2_**L7**]^−^ hydrolyze sarin at 303 K with k_cat_/K_m_ values of 0.051 and 0.11 s^−1^ M^−1^, respectively. DFT calculations confirmed a stepwise associative mechanism with a pentacoordinate phosphoryl intermediate. One of the Zn1-bound alkoxides serves as a general base and deprotonates an incoming water nucleophile which attacks the phosphorus of the Zn2-coordinated substrate. In the catalyst-substrate complex the incoming water molecule hydrogen bonds to Zn-OR and to another alkoxide that bridges Zn1 and Zn2. P-O_w_ bond formation and proton transfer from the water molecule to the terminal alkoxide occur simultaneously. The higher catalytic activity of [Zn_2_**L7**]^−^ was attributed to the higher basicity of the alkoxide groups in **L7** compared to **L5**.

Penkova et al. investigated the hydrolysis of paraoxon by a series of dinuclear pyrazolate complexes (Penkova et al., 2009). The Zn(II)^…^Zn(II) distances in [Zn_2_(**HL34**^**a**^)_2_(pyridine)_2_], [Zn_2_(**HL34**^**b**^)_2_(CH_3_COO)_2_], [Zn_2_(**H**_**2**_**L34**^**c**^)_2_(NO_3_)_2_] and (imH)_2_[Zn_2_(**L34**^**d**^)_2_(H_2_O)_4_] (imH = imidazolium) range from 3.75 to 4.115 Å, i.e., are longer than those in phenoxide-bridged enzyme models and close to the metal-metal distance in alkaline phosphatase (4.0 Å, Stec et al., 2000). Based on the kinetic data and speciation in solution it was proposed that the phosphotriester binds monodentally to one Zn(II) of [Zn_2_(**HL34**^**a**^)_2_(OH)]^−^, while the other Zn(II) provides a metal-bound hydroxide as the nucleophile. [Zn_2_(**L34**^**d**^)_2_(H_2_O)_4_]^2−^ gave a two-fold lower rate acceleration compared to [Zn_2_(**L34**^**a**^)_2_(OH)]^−^, which was attributed to the lack of Zn-OH. The relatively small difference in activity between [Zn_2_(**L34**^**a**^)_2_(OH)]^−^ and [Zn_2_(**L34**^**d**^)_2_(H_2_O)_4_]^2−^ led the authors to the conclusion that Lewis activation is more important for efficient catalysis than metal hydroxide activation. Although the rate constants for the uncatalyzed cleavage of paraoxon and the RNA model HPNP are comparable, the pyrazolate complexes cleave HPNP with a second order rate constant that is about one order of magnitude larger than that for the hydrolysis of paraoxon. This was rationalized with the bridging coordination of the phosphodiester to the dizinc(II) site allowing for double Lewis acid activation.

## Concluding Remarks

It is hoped that this review has shown that the metal catalysis of what appears to be a rather simple chemical reaction has been and continues to be a challenging research question. Model studies using dinuclear zinc(II) complexes have given insight into the possible roles of the Zn^2+^ ions in the dimetallic active sites of phosphatases, the potential effects of metal-substrate interaction on transition state stabilization and the contribution of the different interaction modes to the lowering of the overall energy barrier. In this regard small-molecule biomimetics have proven to be extremely powerful tools. Less has been learned from small-molecule phosphatase models on the actual mechanistic pathway of the natural enzymes, e.g., distinguishing between a monodentate substrate coordination/terminal Zn-OH nucleophile mechanism and a bridging substrate coordination/μ-OH nucleophile mechanism for a specific phosphoesterase and this is not the aim of model studies. There is no universal mechanism for the catalysis of phosphate esters by dinuclear dizinc(II) phosphoesterases. Even when a particular phosphoesterase is considered, there is accumulating evidence that phosphoesterases with a low substrate specificity hydrolyze different substrates by distinct mechanisms.

Small-molecule enzyme models have inherent limitations. They lack the pre-organization of enzymatic sites and the surrounding protein matrix that supports the correct substrate orientation and provides a hydrophobic environment. While the latter has been modeled by non-aqueous media and model systems are increasingly designed to take secondary interactions into account by introducing substituents with hydrogen bonding functionality, it is still (and probably will always be) impossible to mimic the complexity of natural enzymes. Studies in non-aqueous solvents showed a clear effect of the medium on the catalytic activity, but an effect on the mechanism (e.g., A_N_D_N_ vs. A_N_+D_N_) must also be considered. Most experimental studies using small-molecule phosphatase models rely on kinetic data, which highlights the inherent difficulty that the data can support different, kinetically equivalent mechanisms. The use of 4-nitrophenyl esters as DNA and RNA models has been criticized in the literature (Menger and Ladika, 1987) and a few caveats have been pointed out in this review. On the other hand, systematic studies with different phosphate esters have shown changes in the mechanism and the rate-determining step, when the leaving group was changed and thus contributed to a better fundamental understanding of metal-catalyzed phosphate ester hydrolysis.

In contrast to the large amount of data that has been collected on the cleavage of phosphate esters at biomimetic dizinc(II) sites, little attention has been paid to the regeneration of the catalyst. After hydrolysis of the phosphotri-, di- or monoester, the resulting diester, monoester or ortho phosphate will be coordinated in a bridging mode and will bind more strongly to the catalyst as the anionic charge has increased. Ejection of the hydrolysis product would require nucleophilic attack of water on the metal(s). Obviously, the regeneration of the dizinc(II) site is important for catalytic turnover and there is a clear need for future work in this direction.

## Author Contributions

The author confirms being the sole contributor of this work and has approved it for publication.

### Conflict of Interest Statement

The author declares that the research was conducted in the absence of any commercial or financial relationships that could be construed as a potential conflict of interest.
